# Oral Human Papillomavirus in Men Having Sex with Men: Risk-Factors and Sampling

**DOI:** 10.1371/journal.pone.0049324

**Published:** 2012-11-16

**Authors:** Tim R. H. Read, Jane S. Hocking, Lenka A. Vodstrcil, Sepehr N. Tabrizi, Michael J. McCullough, Andrew E. Grulich, Suzanne M. Garland, Catriona S. Bradshaw, Marcus Y. Chen, Christopher K. Fairley

**Affiliations:** 1 Melbourne Sexual Health Centre, Alfred Health, Melbourne, Australia; 2 School of Population Health, University of Melbourne, Melbourne, Australia; 3 Centre for Women’s Health, Gender and Society, University of Melbourne, Melbourne, Australia; 4 Dept of Microbiology and Infectious Diseases, Royal Women’s Hospital, Department of Obstetrics and Gynaecology, University of Melbourne, Murdoch Children’s Research Institute, Melbourne, Australia; 5 Melbourne Dental School, University of Melbourne, Melbourne, Australia; 6 Kirby Institute, University of New South Wales, Sydney, Australia; 7 Dept of Epidemiology and Preventive Medicine, Monash University, Melbourne, Australia; Rollins School of Public Health, Emory University, United States of America

## Abstract

**Background:**

Human papillomavirus (HPV)-associated oropharyngeal squamous cell carcinoma is becoming more common. We examined prevalence and risk factors for oral HPV among men who have sex with men (MSM) and compared sampling and transport methods.

**Methods:**

In 2010, 500 MSM (249 HIV-positive) attending Melbourne Sexual Health Centre answered a questionnaire, swabbed their mouth and throat and collected a gargled oral rinse sample. Half the oral rinse was transported absorbed in a tampon (to enable postage). HPV was detected by polymerase chain reaction, and genotyped by Roche Linear Array®. Men with HPV 16 or 18 were retested after six months.

**Results:**

Any HPV genotype was detected in 19% (95% confidence intervals (CI) 15–25%) of HIV-infected men and 7% (95% CI 4–11%) of HIV-negative men (p<0.001), and HPV 16 was detected in 4.4% (95% CI 2–8%) of HIV-infected men and 0.8% (0.1–2.8%) of HIV-negative men. Oral HPV was associated with: current smoking (adjusted odds ratio (aOR) 2.2 (95%CI: 1.2–3.9)), time since tooth-brushing (aOR per hour 0.87, 95%CI: 0.8–0.96) and number of lifetime tongue-kissing partners aOR 3.2 95%CI: (1.2–8.4) for 26–100 partners and 4.9 95%CI: (1.9–12.5) for>100 partners. Lifetime oral-penile sex partner numbers were significantly associated in a separate model: aOR 2.8(1.2–6.3) for 26–100 partners and 3.2(1.4–7.2) for>100 partners. HPV 16 and 18 persisted in 10 of 12 men after a median six months. Sensitivities of sampling methods compared to all methods combined were: oral rinse 97%, tampon-absorbed oral rinse 69%, swab 32%.

**Conclusions:**

Oral HPV was associated with HIV infection, smoking, recent tooth-brushing, and more lifetime tongue-kissing and oral sex partners. The liquid oral rinse sample was more sensitive than a tampon-absorbed oral rinse or a self-collected swab.

## Introduction

Oncogenic human papillomavirus (HPV), principally genotype 16, is now a recognised cause of a substantial proportion of oropharyngeal squamous cell carcinoma (SCC) and this proportion appears to be increasing. [1], [2], [3] In an Australian cohort, the proportion of HPV-positive oropharyngeal SCC increased from 19% in 1987–1990 to 60% in 2005–6. [4] Over the same period the incidence of non HPV-associated head and neck cancers has been falling. [5], [6].

This increase in HPV-associated oropharyngeal SCC has led to a search for predictors of oral HPV infection in individuals without cancer. Multiple studies indicate that the presence of oral HPV DNA is associated with higher numbers of sexual partners, smoking and HIV infection [7], [8], [9], [10], [11]. However it is unclear whether the risk of HPV infection is determined by the number of sexual partners in one’s lifetime or over a more recent period, and which specific sexual practice carries the greatest risk of infection. It is also unknown how frequently infection occurs after exposure, how long it persists and what determines the duration of infection. In addition, no data are available on the effect of eating, drinking and tooth-brushing on HPV detection.

**Table 1:** Prevalence of oral HPV types, by HIV status, in 500 men who have sex with men.

HPV type	HIV negative N = 251n (%, 95% CI)	HIV positive N = 249n (%, 95% CI)	Prevalence ratio^a^	Overall n (%, 95% CI)
Any HPV type	17 (7, 4–11)	48 (19, 15–25)	2.8	65 (13, 10 −16)
HPV 16	2 (0.8, 0.1–3)	11 (4, 2–8)	5.5	13 (3, 1 −4)
High risk HPV types^b^	5 (2, 0.6–5)	20 (8, 5–12)	4.0	25 (5, 3 −7)
HPV^c^ types 6 or 11 or 16 or 18	5 (2, 0.6–5)	16 (6, 4–10)	3.2	21 (4, 3 −6)
More than 1 type of HPV	3 (0.6, 0.2–3)	18 (7, 4–11)	6.0	21 (4, 3 −6)

CI confidence interval.

(a) Ratio of prevalence in HIV-positive to HIV-negative MSM.

(b) One or more of types 16, 18, 31, 35, 39, 45, 51, 56, 58, 59, 68 which are considered oncogenic in the cervix.

(c) Included in the quadrivalent vaccine.

To better understand the epidemiology of oral HPV infection population-based studies are needed. Population-based studies would be facilitated by self-collected oral samples that could be sent by post to laboratories. Gargled oral rinses have been reported as more sensitive samples for HPV DNA detection than tonsillar and oral mucosal brushings, [10], [12] but large-scale postal surveys involving liquid samples are impractical due to postal regulations in some countries [13].

**Table 2:** Any HPV type detected by oral rinse, tampon-absorbed (TA) oral rinse and self-collected throat-mouth swabs.

	Oral rinse		Oral rinse TA		Positive on any sample		sensitivity
	HPV−	HPV+	HPV−	HPV+	HPV−	HPV+	
Swab HPV−	435	44	449	30	435	44	
Swab HPV+	2	19	6	15	0	21	32% P<0.0001^a^
Oral rinse TA HPV−	437	18	–	–	435	20	
Oral rinse TA HPV+	0	45	–	–	0	45	69% P<0.0001^a^
Oral rinse HPV−	–	–	–	–	435	2	
Oral rinse HPV+	–	–	–	–	0	63	97% P = 0.48^a^

(a) by McNemar’s test for the difference in HPV detection between the specified sampling method and HPV detection on any of the three samples.

We performed a study with two aims. The first was to compare HPV detection from three sampling and transport methods: oral rinse samples, oral rinse samples absorbed in a tampon and a self-collected mouth and throat-mouth swab. The latter two can be mailed. We also assessed the impact of other factors that may affect HPV detection, such as eating, drinking and tooth-brushing.

**Table 3:** Detection of oral HPV analysed by number of reported sexual partners for each sexual practice over three non-overlapping time periods.

Risk factor			HPV detected n(%)	HPV not detectedn	Unadjusted oddsratio (95% CI)	Adjusted^a^ oddsratio (95% CI)
Number of tongue-kissing partners:	*0–2 weeks ago*	0	20 (13)	132	1.0	1.0
		1	29 (15)	169	1.1 (0.6–2.1)	1.3 (0.7–2.6)
		>1	16 (11)	129	0.8 (0.4–1.6)	0.6 (0.3–1.4)
		P trend			0.60	0.34
	*>2–52 weeks ago*	0–1	22 (18)	98	1.0	1.0
		2–9	17 (8)	185	0.4 (0.2–0.8)	0.4 (0.2–0.8)
		≥10	23 (14)	136	0.8 (0.4–1.4)	0.5 (0.3–1.2)
		P trend			0.47	0.08
	*>1 year ago^b^*	0–25	8 (5)	162	1.0	1.0
		26–100	26 (14)	159	3.3 (1.5–7.5)	3.9 (1.7–9.1)
		>100	25 (22)	89	5.7 (2.5–13.1)	7.0 (2.8–17.3)
		P trend			<0.001	<0.001
Number of oral-penile^c^ sex partners:	*0–2 weeks ago*	0	20 (13)	140	1.0	1.0
		1	28 (15)	156	1.3 (0.7–2.3)	1.4 (0.7–2.7)
		>1	17 (11)	135	0.9 (0.4–1.8)	0.5 (0.2–1.1)
		P trend			0.74	0.13
	*>2–52 weeks ago*	0–1	17 (14)	102	1.0	1.0
		2–9	16 (8)	189	0.5 (0.3–1.0)	0.5 (0.2–1.1)
		≥10	29 (18)	132	1.3 (0.7–2.5)	1.5 (0.7–3.2)
		P trend			0.23	0.38
	*>1 year ago^b^*	0–25	14 (7)	180	1.0	1.0
		26–100	25 (16)	135	2.4 (1.2–4.8)	2.2 (1.1–4.6)
		>100	21 (19)	91	3.0 (1.4–6.1)	2.8 (1.3–6.4)
		P trend			0.003	0.005
Number of oral-anal^c^ sex partners:	0–2 weeks ago	0	46 (13)	309	1.0	1.0
		1	10 (10)	91	0.7 (0.4–1.5)	0.6 (0.3–1.4)
		>1	9 (24)	28	2.2 (1.0–4.9)	1.1 (0.4–3.1)
		P trend			0.29	0.56
	*>2–52 weeks ago*	0–1	33 (11)	258	1.0	1.0
		2–9	17 (13)	112	1.2 (0.6–2.2)	0.9 (0.5–1.8)
		≥10	11 (18)	50	1.7 (0.8–3.6)	1.1 (0.4–2.9)
		P trend			0.17	0.73
	*>1 year ago^b^*	0–25	34 (10)	308	1.0	1.0
		26–100	22 (22)	78	2.6 (1.4–4.6)	2.6 (1.3–5.2)
		>100	4 (14)	25	1.4 (0.5–4.4)	1.4 (0.4–5.2)
		P trend			0.02	0.07

CI confidence interval.

(a) For each sexual practice the multivariate model included three variables. These were the number of sexual partners for that sexual practice, in each of the three non-overlapping time periods: previous two weeks, from>2 to 52 weeks ago, more than one year ago.

b) reported number of partners over a lifetime for this sexual practice, minus the reported number for the previous year.

c) Oral sexual practices defined as involving the study participant’s mouth.

Our second aim was to clarify the relative importance of lifetime compared to recent sexual partners and then look at different sexual practices in both time periods. We examined these questions in a population of men who have sex with men (MSM) attending a sexual health centre because they were likely to be at increased risk of oral HPV and do not seem to be protected by the HPV vaccination program which targets women. [14], [15]

**Table 4:** Factors associated with detection of HPV in the oropharynx.

Risk factor	HPV detectedn(%)	HPV not detected n	Unadjusted oddsratio (95% CI)	Model 1. Adjusted^a^odds ratios (95% CI)	Model 2. Adjusted^b^odds ratios (95% CI)
Age (OR per year)			1.03 (1.01–1.05)	1.02 (0.99–1.05)	1.02 (1.00–1.05)
HIV negative	17 (7)	234	1.0	1.0	1.0
HIV positive	48 (19)	201	3.3 (1.8–5.9)	2.3 (1.1–4.7)	2.1 (1.0–4.2)
Non-smoker	33 (9)	317	1.0	1.0	1.0
Current smoker	32 (21)	118	2.6 (1.5–4.4)	2.2 (1.2–3.9)	2.1 (1.2–3.7)
Hours since brushed teeth (OR per hour)			0.86 (0.8 −0.95)	0.87 (0.8–0.96)	0.87 (0.8–0.96)
Anogenital warts never	29 (9.3)	283	1.0	1.0	1.0
Anogenital warts ever	36 (19.1)	152	2.3 (1.4–3.9)	1.8 (0.98–3.2)	1.9 (1.0–3.3)
Ejaculation in mouth by<half lifetime oral-penile sex partners	45 (11.1)	361	1.0	1.0	1.0
Ejaculation in mouth by ≥half lifetime oral-penile sex partners	20 (23.0)	67	2.4 (1.3–4.3)	1.9 (0.99–3.7)	1.8 (0.9–3.5)
0–25 tongue-kissing partners in lifetime	6 (4)	131	1.0	1.0	
26–100 tongue-kissing partners in lifetime	22(12)	166	2.9 (1.1–7.3)	3.2 (1.2–8.4)	
>100 tongue-kissing partners in lifetime	31(21)	119	5.7 (2.3–14.1)	4.9 (1.9–12.5)	
0–25 oral-penile^c^ sex partners in lifetime	9(5)	163	1.0		1.0
26–100 oral-penile^c^ sex partners in lifetime	24(15)	141	3.1 (1.4–6.9)		2.8 (1.2–6.3)
>100 oral-penile^c^ sex partners in lifetime	27(19)	114	4.3 (1.9–9.5)		3.2 (1.4–7.2)
Chi-squared for model (degrees of freedom)				49.6 (6)	45.9 (6)
					

CI confidence interval.

(a) Odds ratios (OR) adjusted for age, HIV status, current smoking, time since last brushed teeth and number of lifetime tongue-kissing partners.

(b) Odds ratios (OR) adjusted for age, HIV status, current smoking, time since last brushed teeth and number of lifetime oral-penile sex partners.

(c) Oral sex defined as only involving the study participant’s mouth.

## Methods

This was a cross-sectional study of HIV-positive and HIV-negative MSM attending Melbourne Sexual Health Centre, Victoria, Australia, between 2 March 2010 and 17 June 2010. Methods for the two components of the study are described separately.

**Table 5:** HPV results in 37 HIV positive men from this study, who were tested for anal HPV in another ongoing study after a mean of 16 months.

HPV type and site	HPV negative (n)	HPV positive (n)	% HPV positive (95%CIl)
Any type - anal	2	35	95 (87–100)
Any type - oral	31	6	16 (6–32)
Type 16 - anal	24	13	35 (19–51)
Type 16 - oral	36	1	3 (0–8)

CI confidence interval.

### Risk Factor Study

In the clinic, participants completed a written questionnaire about risk factors including sexual history, smoking, alcohol-consumption and genital warts. The questionnaire also asked about factors that may affect detection, including how long prior to specimen collection they last ate, drank or brushed their teeth. The sexual history covered their number of sexual partners over different time periods (last two weeks, last 12 months, and lifetime) for tongue-kissing, oral-penile sex, and oral-anal sex. Each of these sexual practices was defined as involving only the participant’s mouth. Participants were also asked how many days since they had each type of sex, the proportion of lifetime male oral sex partners who ejaculated in their mouths, and the proportion who used condoms during oral sex with the study participant.

### Sampling Study

After providing written informed consent, participants were shown a video describing how to swab their own gums, mouth and throat with a flocked swab (Copan Diagnostic, Brescia, Italy). This was agitated in RNA stabilization reagent (RNAlater, Ambion Inc, Austin, USA) and transported to the laboratory for processing. Participants also gargled 20 ml saline for ten seconds and expelled it into a container (oral rinse sample). A researcher then divided the oral rinse sample into equal halves: one was absorbed by a tampon (Stayfree® Meds, Johnson & Johnson, Australia) while the other was left in the original container. In the laboratory, the tampon was squeezed firmly to retrieve as much absorbed saline as possible. The order of collecting the oral rinse and throat swab samples from participants was alternated weekly.

DNA was extracted using MagNA Pure LC (Roche Molecular Systems, Alameda, CA, USA). A 20 µl aliquot of extracted DNA was amplified in a PGMY09/11-based HPV consensus PCR assay [16], with a PCR-ELISA detection protocol. [17] All assays incorporated amplification of the β-globin gene as an internal control. All samples positive on the PGMY09/11 PCR test were genotyped using HPV Linear Array® (LA) Genotyping Test (Roche Molecular Systems), using 50 µl of extracted DNA, and following the manufacturer’s instructions with minor modifications as previously reported. [18], [19] LA identifies 37 genotypes: 6, 11, 16, 18, 26, 31, 33, 35, 39, 40, 42, 45, 51, 52, 53, 54, 55, 56, 58, 59, 61, 62, 64, 66, 67, 68, 69, 70, 71, 72, 73, 81, 82 (previously known as IS39), 83, 84 and 89 (previously known as CP6108) [20].

Negative and positive controls were processed with each run, and lack of signal in the negative control was used to monitor possible carryover.

Men who had HPV 16 or 18 detected on any sample were referred for oral examination and retesting for oral HPV using a gargled oral rinse and a swab taken by an oral medicine specialist, six months later. A subset of 37 HIV positive men had anal swabs in a subsequent study of anal cancer screening, performed 16 months after this study. This study recruited from the same HIV-positive MSM population with the exception that only men aged ≥35 years were eligible. These swabs were tested for HPV by the same method and these data are included for comparison.

Sample sensitivity was calculated by comparing the number of positive specimens by each method to the total positive by any method and McNemar’s test was applied. HPV prevalence estimates and 95% confidence intervals (CI) were calculated using exact methods. Unadjusted and adjusted odds ratios and 95%CI were calculated to investigate associations with HPV by logistic regression using Stata 11.2 (Statacorp, College Station, Texas).

The questionnaire recorded number of sexual partners over three overlapping time intervals: the last 2 weeks, 12 months or lifetime. In order to select the most appropriate of these for the multivariate model we created three variables of non-overlapping time periods: variable 1) number of partners in the last 2 weeks; variable 2) number of partners in the last 12 months minus the number of partners in the last 2 weeks; and variable 3) number of partners over the lifetime minus the number of partners in the last 12 months. This was done for each of the sexual practices: tongue-kissing, oral-penile sex and oral-anal sex.

A separate model was generated for each sexual practice which included only the above three variables. This measured the association with oral HPV of each non-overlapping time period, adjusted for the other time periods.

Other risk-factors associated with HPV at P≤0.05 in the crude analysis were included in the adjusted models. Using a stepwise forward approach, variables were removed from the model as they became non-significant, with the exception of age. Because of the strong correlation between number of partners for oral-penile sex and tongue-kissing and the strong association of each with HPV, two separate logistic regression models are presented.

With an overall HPV prevalence of 13%, power of 80% and significance of 5%, a sample size of 500 would allow us to detect an odds ratio of 2.2 for a risk factor present in 30% of controls.

### Ethics Statement

This research was approved by the Alfred Health Human Ethics Committee.

## Results

We recruited 500 MSM of whom 249 were HIV-positive and 251 HIV-negative. Of those approached to participate, 94% of HIV-positive men and 97% of HIV negative men agreed to participate. The median age of participants was 37 years, intraquartile range (IQR 27–45 years).

Sixty five men (13%; 95%CI: 10–16%) had at least one HPV type: 13 (3%; 95%CI: 1–4%) had HPV 16, 2 (0.4%; 95%CI:0.05–1.4%) had HPV 18, 21 (4%;95%CI 3–6%) had more than one genotype detected (range 2–7 types), and 21 (4%;95%CI: 3–6%) had at least one of the vaccine-preventable genotypes (6,11,16,18). Of the 251 HIV negative men, 17 (7% 95% CI: 4–11%) had at least one HPV type compared to 48 (19% 95%CI: 15–25%) of the 249 HIV-positive men, p<0.001 (Table 1).

### Sensitivity of Sampling Methods

Of the 65 samples positive on any of the three sampling methods, the number positive for each method was: oral rinse 63 (sensitivity 97%, 95%CI: 89–100%), tampon-absorbed oral rinse 45 (sensitivity 69%, 95%CI: 57–80%) and swab 21 (sensitivity 32%, 95%CI: 21–45%) (Table 2). The order in which the samples were collected did not influence the proportion of swabs or rinses that were positive for at least one type of HPV (P = 0.93).

### Risk Factor Analysis

Table three shows the crude odds ratios for the three sexual behaviours (tongue-kissing, oral-penile sex, and oral-anal sex) over the three non-overlapping time periods (0–2 weeks,>2 weeks to one year, one year to lifetime) and only the number of sexual partners more than a year ago was significantly associated with HPV detection.

Table three also shows, for each sexual practice, the adjusted odds ratios when all three time periods are included in the model. Only the number of partners more than a year ago was significantly associated with HPV detection, and only for tongue-kissing and oral-penile sex (Table 3).

Table four shows crude odds ratios for factors significantly associated with HPV detection. These were HIV infection, older age, more recent brushing of teeth, current smoking, ever having anogenital warts, and ejaculation occurring more commonly with oral sex (Table 4).

HPV detection was not associated with: the number of days since last oral-penile sex (P = 0.04) or tongue-kissing (P = 0.4), hours since last ate (P = 0.4) or drank (P = 0.5), whether or not condoms were used for half or more oral-penile sex partners (P = 0.9), current or nadir CD4 T cell count (P>0.3 for both) or HIV viral load (P = 0.5) (if HIV infected. Data not shown).

Table four shows the adjusted odds ratios in two logistic regression models. The first model includes lifetime number of tongue-kissing partners, and the second includes lifetime number of oral-penile sex partners. These two practices were strongly correlated (chi-squared p<0.001). In both models, HPV detection was significantly associated with current smoking, HIV infection, more recent brushing of teeth and the lifetime number of sexual partners for either tongue-kissing or oral-penile sex.

### Follow-up Data

Twelve of the 13 men with HPV 16 or 18 were retested after a median of six months (185 days, range 139–211) and 10 of the 12 (83%) remained positive for the same genotype.

Of the 249 HIV positive men, 37 were involved in a subsequent ongoing study of HIV-positive MSM aged ≥35 years (personal communication TRH Read) where anal samples were taken a mean of 16 months after the oral samples. Of these 37 men 35, 95%(95% CI 87%–100%) tested positive for anal HPV. Of the 13 with anal HPV 16, 35%(95% CI 19%–51%), only one had oral HPV 16 detected (Table 5).

## Discussion

In our study of sampling and transport methods, oral rinse samples were significantly more sensitive than self-collected swabs and absorbing the oral rinse into a tampon for postage resulted in a significantly lower rate of oral HPV detection. HPV detection was more likely in those who had recently brushed their teeth and in current smokers. A higher number of lifetime sexual partners for tongue kissing and oral-penile sex, also predicted oral HPV detection. However only a minority of men with high numbers of oral sex partners were HPV positive, and prevalence in the mouth was much lower than prevalence in the anus, which was high in this and other studies of MSM. [14], [21] The same genotype of HPV was detected in 83% of the 12 men retested six months later. Together these data suggest that oral HPV infection may be difficult to acquire, but once present may persist many years.

The finding that recent tooth-brushing increases HPV detection also suggests that current sampling techniques may be improved by prior epithelial abrasion, similar to that used for anogenital HPV detection in men. [22] The likelihood of detecting oral HPV fell in a linear fashion by about 14% with each additional hour after brushing teeth, suggesting that abrasion of oral mucosa improves collection of infected cells in an oral rinse. D’Souza and coworkers have reported an association between oropharyngeal cancer and infrequent toothbrushing, but this has not been reported for HPV detection and may be related to a different causal pathway for oropharyngeal cancer. [23] These investigators have combined oropharyngeal brushings with oral rinses [7], [23] and have shown higher detection in oral rinses than in brushings [10] but there are no reports comparing HPV detection in oral rinse samples with and without prior abrasion. Oral HPV detection was also associated with current smoking and this also has been reported by D’Souza. [7], [8] Smoking causes oral epithelial thickening and periodontal disease [24] and given our observation that epithelial abrasion increases HPV detection, it may be that the epithelial effects of smoking contribute to increased detection in smokers.

Reports differ on whether oral HPV is significantly associated with recent or lifetime numbers of sexual partners [9], [10], [25]. There are three potential reasons for these differences. Firstly, if only young adults within a few years of onset of sexual activity are studied, recent partners may approximate lifetime partners. This explanation is suggested by the observation that oral HPV is significantly associated with recent partners in studies that involve only young adults (age<24 [7] or<27 [26]). Secondly, most studies are of women and heterosexual men with fewer partners than the MSM population assessed in the present study. Finally, we tested the association of oral HPV with partner numbers in non-overlapping time periods, whereas other investigators have not separated them.

We found that HPV detection rose with age, consistent with most other studies [9], [10], [25] and importantly no studies have shown significantly declining prevalence with age. An increasing prevalence of HPV with age is consistent with the finding that lifetime, but not recent partner numbers, are most strongly associated with oral HPV detection suggesting that once acquired, oral HPV infection persists a long time. However if this is true the relatively low prevalence of HPV infection, despite a high numerical sexual exposure can only be explained if oral HPV infection is difficult to acquire. These findings contrast with cervical HPV infection which is acquired rapidly after commencing sexual activity, is related to recent sexual partners and its prevalence falls with age. [8], [27], [28]

Our study had a number of limitations. Firstly, it was cross-sectional and the associations are subject to the limitations of this design, such as unmeasured or incomplete adjustment for confounding. Secondly, the participants were from one sexual health service and therefore it may not be reasonable to generalise these findings to populations with a lower sexual risk. Finally, the reported number of sexual partners may have been affected by recall or social desirability bias.

Future studies could examine the effect of brushing prior to obtaining oral rinse samples to enhance HPV detection. We found a high level of HPV persistence in the small number of men tested after six months. Larger longitudinal studies are required to confirm this and to establish the age of acquisition of oral HPV to inform future vaccination policies.
